# Comprehensive autoantibody profiling in systemic autoimmunity by a highly-sensitive multiplex protein array

**DOI:** 10.3389/fimmu.2023.1255540

**Published:** 2023-08-28

**Authors:** Ai Kuzumi, Yuta Norimatsu, Kazuki M. Matsuda, Chihiro Ono, Taishi Okumura, Emi Kogo, Naoki Goshima, Takemichi Fukasawa, Natsumi Fushida, Motoki Horii, Takashi Yamashita, Asako Yoshizaki-Ogawa, Kei Yamaguchi, Takashi Matsushita, Shinichi Sato, Ayumi Yoshizaki

**Affiliations:** ^1^ Department of Dermatology, Graduate School of Medicine, University of Tokyo, Tokyo, Japan; ^2^ ProteoBridge Corporation, Tokyo, Japan; ^3^ Molecular Profiling Research Center for Drug Discovery, National Institute of Advanced Industrial Science and Technology, Tokyo, Japan; ^4^ Department of Clinical Cannabinoid Research, Graduate School of Medicine, University of Tokyo, Tokyo, Japan; ^5^ Department of Dermatology, Faculty of Medicine, Institute of Medical, Pharmaceutical and Health Sciences, Kanazawa University, Kanazawa, Japan

**Keywords:** systemic sclerosis, polymyositis, dermatomyositis, autoantibody, proteomics

## Abstract

Comprehensive autoantibody evaluation is essential for the management of autoimmune disorders. However, conventional methods suffer from poor sensitivity, low throughput, or limited availability. Here, using a proteome-wide human cDNA library, we developed a novel multiplex protein assay (autoantibody array assay; A-Cube) covering 65 antigens of 43 autoantibodies that are associated with systemic sclerosis (SSc) and polymyositis/dermatomyositis (PM/DM). The performance of A-Cube was validated against immunoprecipitation and established enzyme-linked immunosorbent assay. Further, through an evaluation of serum samples from 357 SSc and 172 PM/DM patients, A-Cube meticulously illustrated a diverse autoantibody landscape in these diseases. The wide coverage and high sensitivity of A-Cube also allowed the overlap and correlation analysis between multiple autoantibodies. Lastly, reviewing the cases with distinct autoantibody profiles by A-Cube underscored the importance of thorough autoantibody detection. Together, these data highlighted the utility of A-Cube as well as the clinical relevance of autoantibody profiles in SSc and PM/DM.

## Introduction

Autoantibodies represent a breakdown of self-tolerance and are the hallmarks of autoimmunity (1). Accumulating evidence also suggests that autoantibodies are closely linked to the pathogenesis, progression, and prognosis of a number of autoimmune disorders (2). In particular, various autoantibodies have been identified in connective tissue diseases such as systemic sclerosis (SSc) and polymyositis/dermatomyositis (PM/DM), where patients are classified into distinct clinical phenotypes based on autoantibody profiles (3, 4). Therefore, precise characterization of autoantibodies is important not only for diagnosis but also for the proper management of these diseases.

While immunoprecipitation (IP) is considered the gold standard for detecting autoantibodies, its use is restricted to a few specialized laboratories because of the cumbersome procedures (5). To overcome this limitation, immunoblotting assays have been developed as simpler alternatives for autoantibody testing. However, some autoantigens are susceptible to protein degradation and lose reactivity in these assays, leading to a high rate of false negatives (6, 7). Enzyme-linked immunosorbent assays (ELISAs) detect autoantibodies with high sensitivity, but are available only for a limited number of autoantibodies (3, 4). Moreover, conventional ELISAs suffer from low throughput and are not suitable for simultaneous evaluation of multiple autoantibodies. Thus, there is a great need for the development of multiplex autoantibody assays with high reliability and availability.

Recent technological advances in proteomics now allow high-throughput protein expression *in vitro* on a whole-proteome scale (8–10). We have previously described a comprehensive wet protein array, in which more than 19,000 proteins from a proteome-wide human cDNA library (HuPEX) are expressed *in vitro* under humidity control to prevent their degradation (11). Here, using this method, we developed a novel multiplex protein array (autoantibody array assay; A-Cube) covering 65 target antigens of 43 autoantibodies that are associated with SSc and PM/DM. The assay performance was validated against IP and established ELISA, supporting its use in clinical and research settings. Further, through a comprehensive autoantibody profiling of 357 SSc and 172 PM/DM patients by the assay, we uncovered a diverse landscape of autoantibodies with their clinical implications in these diseases.

## Methods

### Patients

Serum samples were obtained from Japanese patients with SSc (n = 357) and PM/DM (n = 172). All SSc patients fulfilled the ACR/EULAR classification criteria (12), and all PM/DM patients met the Bohan and Peter criteria (13, 14). No patients fulfilled the Sontheimer criteria for clinically amyopathic DM (15) or were clinically suspected of having statin-induced myositis (16). In addition, 93 healthy Japanese individuals were included as controls. The study was conducted in accordance with the Declaration of Helsinki and approved by the ethics committee of the University of Tokyo Graduate School of Medicine. Written informed consent was obtained from all participants.

### *In vitro* protein expression

**Figure 1:**
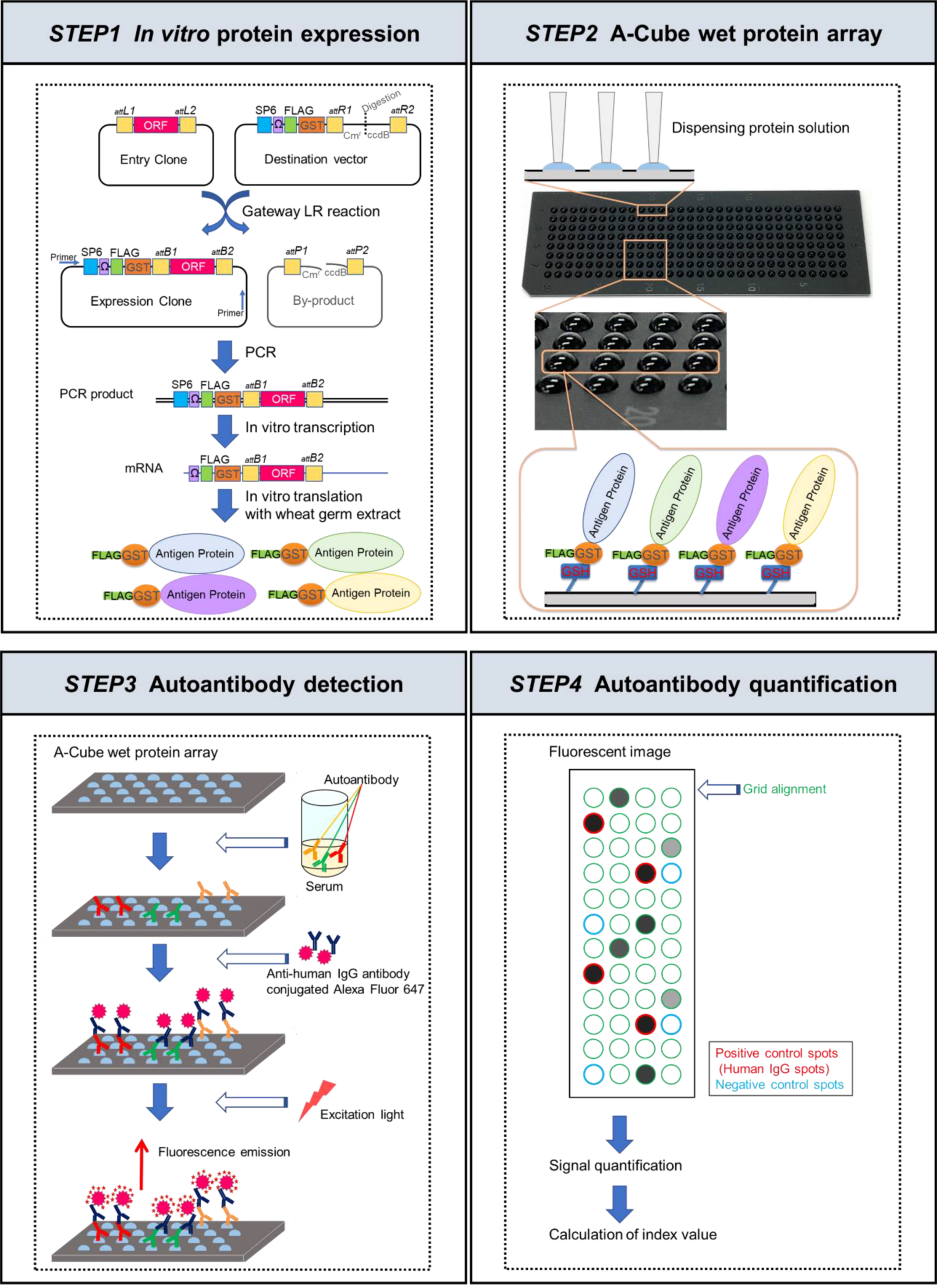
Schematic figures of A-Cube. In the first step, proteins were synthesized *in vitro* from the proteome-wide human cDNA library (HuPEX) by the wheat germ cell-free synthesis system. In the second step, A-Cube array was produced. In the third step, autoantibody detection was performed using human serum samples. In the fourth step, autoantibody quantification was performed.

The overview of the workflow is presented in **Figure 1**. Sixty-five antigens of 43 autoantibodies associated with SSc and PM/DM were selected (**Table 1**). *In vitro* protein expression was used a wheat germ cell-free translation system (8–10). Target clones of antigens in HuPEX entry clone library (17) were recombined into a destination/expression vector pEW-5FG for producing N-terminal FLAG-GST-tag proteins with GATEWAY cloning system (Thermo Fisher Scientific, Waltham, MA, USA). To synthesize the target antigens, the transcription unit on the vector was amplified by PCR and used for an *in vitro* transcription, followed by a wheat germ cell-free translation system (FASMAC, Kanagawa, Japan) by using the method of previous paper (17, 18).

**Table 1:** Autoantibodies and their target antigens detected by A-Cube.

Antibody	Antigen	SSc	PM/DM	Ctrl
SSc-associated autoantibodies				
CENP-A	CENPA	110/357 (30.8%)	9/172 (5.2%)	0/93 (0.0%)
CENP-B	CENPB	115/357 (32.2%)	12/172 (7.0%)	0/93 (0.0%)
CENP-C	CENPC	96/357 (26.9%)	7/172 (4.1%)	0/93 (0.0%)
Topoisomerase I	TOP1	96/357 (26.9%)	0/172 (0.0%)	0/93 (0.0%)
RNA polymerase III (RPC155)	POLR3A	40/357 (11.2%)	1/172 (0.6%)	0/93 (0.0%)
RNA polymerase III (RPC62)	POLR3C	26/357 (7.3%)	1/172 (0.6%)	0/93 (0.0%)
RNA polymerase I	POLR1A	6/357 (1.7%)	1/172 (0.6%)	0/93 (0.0%)
RNA polymerase II	POLR2A	8/357 (2.2%)	1/172 (0.6%)	0/93 (0.0%)
Th/To	POP1	6/357 (1.7%)	0/172 (0.0%)	0/93 (0.0%)
	RPP25	0/357 (0.0%)	0/172 (0.0%)	0/93 (0.0%)
U3-RNP	FBL	7/357 (2.0%)	1/172 (0.6%)	0/93 (0.0%)
NOR90	UBTF	9/357 (2.5%)	2/172 (1.2%)	1/93 (1.1%)
U11/U12-RNP	RNPC3	2/357 (0.6%)	1/172 (0.6%)	0/93 (0.0%)
SSSCA1	SSSCA1	7/357 (2.0%)	1/172 (0.6%)	0/93 (0.0%)
AMA-M2	DLAT	29/357 (8.1%)	2/172 (1.2%)	0/93 (0.0%)
	DLST	3/357 (0.8%)	0/172 (0.0%)	0/93 (0.0%)
	DBT	21/357 (5.9%)	8/172 (4.7%)	1/93 (1.1%)
	PDHX	9/357 (2.5%)	1/172 (0.6%)	2/93 (2.2%)
p80-coilin	COIL	5/357 (1.4%)	2/172 (1.2%)	4/93 (4.3%)
PM/DM-associated autoantibodies				
Jo-1	HARS	0/357 (0.0%)	9/172 (5.2%)	0/93 (0.0%)
PL-7	TARS	2/357 (0.6%)	11/172 (6.4%)	0/93 (0.0%)
PL-12	AARS	1/357 (0.3%)	6/172 (3.5%)	0/93 (0.0%)
EJ	GARS	1/357 (0.3%)	7/172 (4.1%)	0/93 (0.0%)
KS	NARS	0/357 (0.0%)	7/172 (4.1%)	0/93 (0.0%)
OJ	IARS	0/7	2/172 (1.2%)	0/93 (0.0%)
	EPRS	0/7	4/35	0/10
	LARS	0/7	6/35	0/10
	MARS	0/7	2/35	0/10
	QARS	0/7	5/35	0/10
	KARS	0/7	0/35	0/10
	RARS	0/7	0/35	0/10
	DARS	0/7	1/35	0/10
	AIMP1	0/7	3/4	0/10
	AIMP2	0/7	0/4	0/10
	AIMP3	0/7	0/4	0/10
Zo	FARSA	1/357 (0.3%)	1/172 (0.6%)	0/93 (0.0%)
	FARSB	0/357 (0.0%)	0/172 (0.0%)	0/93 (0.0%)
Ha	YARS	0/7	0/69	0/10
SRP	SRP54	0/357 (0.0%)	3/172 (1.7%)	0/93 (0.0%)
	SRP14	0/357 (0.0%)	0/172 (0.0%)	0/93 (0.0%)
	SRP19	0/357 (0.0%)	1/172 (0.6%)	0/93 (0.0%)
	SRP68	0/357 (0.0%)	1/172 (0.6%)	0/93 (0.0%)
	SRP72	0/357 (0.0%)	1/172 (0.6%)	0/93 (0.0%)
Mi-2	CHD3	0/357 (0.0%)	8/172 (4.7%)	0/93 (0.0%)
	CHD4	0/357 (0.0%)	9/172 (5.2%)	0/93 (0.0%)
TIF1-γ	TRIM33	0/357 (0.0%)	42/172 (24.4%)	0/93 (0.0%)
TIF1-α	TRIM24	1/357 (0.6%)	31/172 (18.0%)	0/93 (0.0%)
TIF1-β	TRIM28	0/357 (0.0%)	3/172 (1.7%)	0/93 (0.0%)
MJ (NXP-2)	MORC3	0/357 (0.0%)	6/172 (3.5%)	0/93 (0.0%)
SAE	SAE1	0/25	2/69	0/10
	UBA2	0/17	1/39	0/10
SMN	SMN1	4/357 (1.1%)	5/172 (2.9%)	0/93 (0.0%)
cN1A	NT5C1A	2/25	8/69	0/10
overlap syndrome-associated autoantibodies				
U1-RNP_70	SNRNP70	12/357 (3.4%)	5/172 (2.9%)	0/93 (0.0%)
U1-RNP_A	SNRPA	13/357 (3.6%)	4/172 (2.3%)	0/93 (0.0%)
U1RNP_C	SNRPC	12/357 (3.4%)	4/172 (2.3%)	0/93 (0.0%)
U2RNP	SNRPB2	9/357 (2.5%)	5/172 (2.9%)	1/93 (1.1%)
Ku	XRCC5	1/357 (0.6%)	1/172 (0.6%)	0/93 (0.0%)
	XRCC6	3/357 (0.8%)	1/172 (0.6%)	0/93 (0.0%)
PM-Scl100	EXOSC10	1/357 (0.6%)	0/172 (0.0%)	2/93 (2.2%)
PM-Scl75	EXOSC9	0/357 (0.0%)	1/172 (0.6%)	0/93 (0.0%)
Ki	PSME3	5/357 (1.4%)	4/172 (2.3%)	0/93 (0.0%)
SS-A/Ro52	TRIM21	90/357 (25.2%)	62/172 (36.0%)	3/93 (3.2%)
SS-A/Ro60	TROVE2	66/357 (18.5%)	19/172 (11.0%)	0/93 (0.0%)
SS-B	SSB	14/357 (3.9%)	5/172 (2.9%)	0/93 (0.0%)

Data were presented as No. (%). Some autoantibodies were measured in part of the cohort after they were added to the assay.

### A-Cube wet protein array

The synthesized proteins were captured on array plates under wet conditions. For preparing the array plates, amino group-modified glass plates (SDM0011, Matsunami Glass, Osaka, Japan) were coated with 50 mM glutathione (GSH) via Sulfo-SMPB (22317, Thermo Fisher Scientific). The translation reaction mixture containing the FLAG-GST-tagged target protein was diluted 5 times with PBS and simultaneously spotted onto 4 GSH-coated glass plates (240 spots/plate) using a 1536-channel independent cylinder system (BIOTEC, Tokyo, Japan). The translation reaction mixture was spotted in duplicate. After spotting, the plates were incubated at room temperature for 30 min and washed with Tris-buffered saline containing 0.1% Tween 20 (TBST; 9997S, Cell Signaling Technology, Danvers, MA, USA). The plates were then incubated in blocking buffer (50 mM HEPES pH 7.5, 200 mM NaCl, 0.08% Triton-X, 25% Glycerol, 5 mM GSH, 0.3% skim milk, and 1 mM DTT) and stored at −80˚C until use. The autoantibody assay using this array plate was named A-Cube.

### Autoantibody detection

The blocking buffer on the plates was thawed at room temperature and discarded. The plates were treated with serum diluted 3:1000 in PBS containing 0.1% skim milk, 1x Synthetic Block (PA017, Invitrogen, Waltham, MA, USA), and 0.1% Tween 20. The amount of serum needed per test was 300 µL. After the reaction at room temperature for 1 h, the plates were washed 3 times with TBST and incubated with goat anti-human IgG (H+L) Alexa Flour^®^ 647 conjugate (A-21445, Invitrogen) diluted 1:1000 in PBS containing 1x Synthetic Block and 0.1% Tween 20 at room temperature for 1 h. Then the plates were washed twice with TBST, washed again with running reverse osmosis water, and air-dried. Finally, the plates were scanned at a 100-µm resolution using a GenePix 4000B Microarray Scanner (Molecular Devices, San Jose, CA, USA). The scanned images were saved as 16-bit tiff files. Array-Pro Analyzer ver. 6.3.1 (Media Cybernetics, Rockville, MA, USA) was used to determine the median value within each spot for signal quantification. The negative control spots were prepared using distilled water (10977015, Invitrogen) instead of mRNA during protein preparation. The positive control spots were prepared using mRNA encoding human IgG for protein synthesis.

### Autoantibody quantification

The autoantibody quantification was performed based on the fluorescent values obtained from reactions of serum with the protein spots. The level of each autoantibody was calculated as below:


$$Index\;value\;=\frac{\;F_{autoantigen}\;-F_{negative\;control}}{F_{positive\;control}\;-F_{negative\;control}}\;\times 100$$

$$F_{autoantigen}:\text{ Fluorescent intensity of autoantigen spot}$$

$$F_{negative\;control}:\text{ Fluorescence intensity of negative control spot}$$

$$F_{positive\;control}:\text{ Fluorescence intensity of positive control spot}$$

The cut-off value of each autoantigen was set as follows, based on the mean and SD of the healthy controls: positive (> 25 for SRP14, SRP19, SRP68, SRP72, CHD3, CHD4, and NT5C1A; >13 for CENPA, POLR2A, DLST, DBT, COIL, TRIM21, and SMN1; and > 10 for the other autoantigens), negative (< 10 for SRP14, SRP19, SRP68, SRP72, CHD3, CHD4, NT5C1A, CENPA, POLR2A, DLST, DBT, COIL, TRIM21, and SMN1;< 7 for the other autoantigens), equivocal (10–25 for SRP14, SRP19, SRP68, SRP72, CHD3, CHD4, and NT5C1A; 10–13 for CENPA, POLR2A, DLST, DBT, COIL, TRIM21, and SMN1; 7–10 for the other autoantigens). The reproducibility of the assay was supported by the consistent results of two independent measurement using randomly selected 10 serum samples.

### ELISA

In addition to A-Cube, serum levels of anti-topoisomerase I antibody, anti-CENP-B antibody, anti-RNA polymerase III (RPC155) antibody, anti-U1-RNP antibody, anti-ARS antibody, anti-Mi-2 antibody, and anti-TIF1-γ antibody were measured using commercially-available ELISA kits (MESACUP-3 test Scl-70, MESACUP-2 test CENP-B, MESACUP anti-RNA polymerase III test, MESACUP-2 test RNP, MESACUP anti-ARS test, MESACUP anti-Mi-2 test, and MESACUP anti-TIF1-γ test; MBL, Nagoya, Japan). The experiments were performed in accordance with the manufacturer’s instructions.

### Fluoroenzyme immunoassay

In addition to A-Cube, serum levels of anti-AMA M2 antibody were measured using fluoroenzyme immunoassay (FEIA) kits (14-5649-01, Phadia, Freiburg, Germany) according to the manufacturer’s instruction.

### IP assays

IP assays were performed with K562 cell extract, as previously described (5). Briefly, 10 µl of serum was mixed with 2 mg of protein-A Sepharose beads (GE17-0780-01, GE Healthcare, Chicago, IL, USA) in 500 µl of IP buffer (10 mM Tris-HCl pH 8.0, 50 mM NaCl, 0.1% Nonidet P-40), incubated for 2 h at 4˚C, and washed 5 times with IP buffer. Antibody-coated Sepharose beads were then mixed with 100 µl of [^35^S] methionine-labelled K562 cell extract and rotated at 4°C for 2 h. After 5 washes, the beads were resuspended in sodium dodecyl sulfate (SDS) sample buffer (70607, Millipore, Burlington, MA, USA), and the polypeptides were fractionated by 7.5% SDS-polyacrylamide gel electrophoresis. Labelled proteins were analyzed by autoradiography. IP assays were performed and analyzed blindly to the results of A-Cube.

### Indirect immunofluorescence

Indirect immunofluorescence tests were performed on monolayer HEp-2 cells (4645, MBL) in accordance with the manufacturer’s instruction. All slides were evaluated blindly and independently by AK and AY.

### Statistical analysis

The correlation between continuous variables was analyzed by Spearman correlation test. The concordance between A-Cube and ELISA/FEIA kits was calculated and expressed as Cohen’s kappa coefficient. P values of< 0.05 were considered statistically significant. GraphPad Prism 7.03 was used for statistical analysis.

## Results

### Autoantibody detection in human serum samples

**Figure 2:**
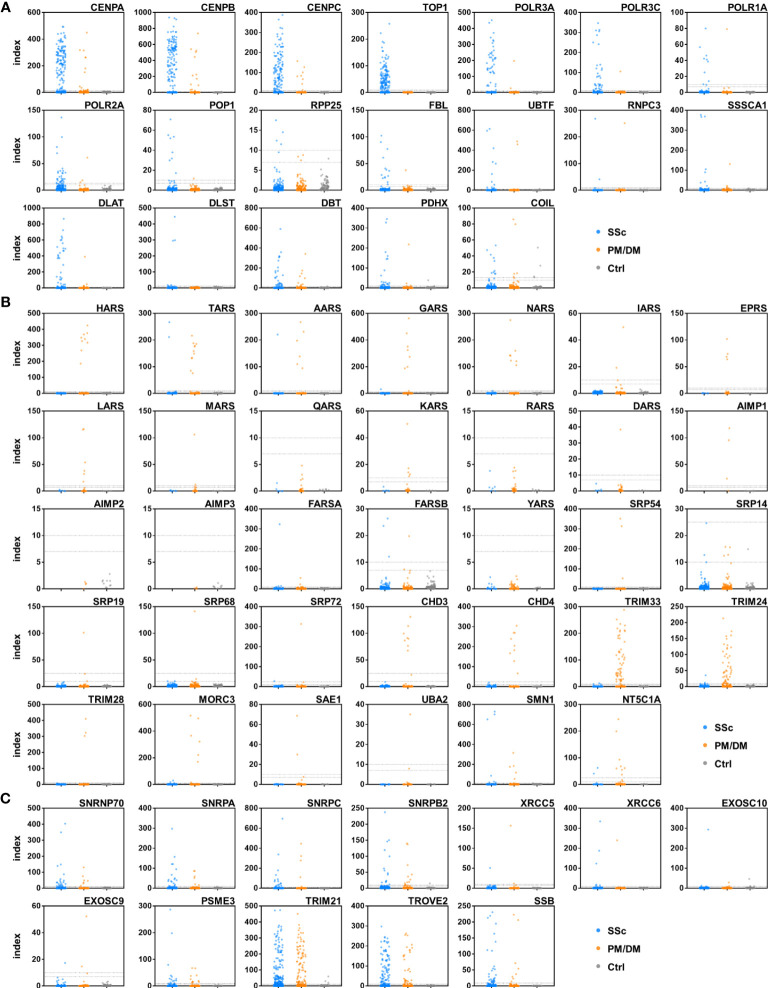
Autoantibody detection in human serum samples. Serum samples were incubated with target antigens of autoantibodies associated with SSc **(A)**, PM/DM **(B)**, and overlap syndrome **(C)**. Data points were jittered horizontally to improve their visibility. Dotted lines indicate the cut-offs for positive/equivocal/negative results. PM/DM, polymyositis/dermatomyositis; SSc, systemic sclerosis.

A total of 622 serum samples from 357 SSc patients, 172 PM/DM patients, and 93 healthy controls were analyzed by A-Cube (**Table 1**; **Figure 2**). Among SSc-associated autoantibodies, anti-CENP-B antibody, anti-topoisomerase I antibody, and anti-RNA polymerase III (RPC155) antibody were commonly detected in SSc patients, with the prevalence rate of 32.2%, 26.9%, and 11.2%, respectively. Among PM/DM-associated autoantibodies, anti-transcriptional intermediary factor 1-γ (TIF1-γ) antibody was most commonly detected in 24.4% of PM/DM patients, followed by anti-TIF1-α antibody (18.0%) and anti-PL-7 antibody (6.4%). Overlap syndrome-associated autoantibodies were mostly detected in both SSc and PM/DM patients, and among them, anti-SS-A/Ro52 antibody had the highest prevalence rate of 25.2% in SSc and 36.0% in PM/DM. Most of the autoantibodies were not detected in healthy controls.

### Comparison with established ELISA and FEIA kits

**Figure 3:**
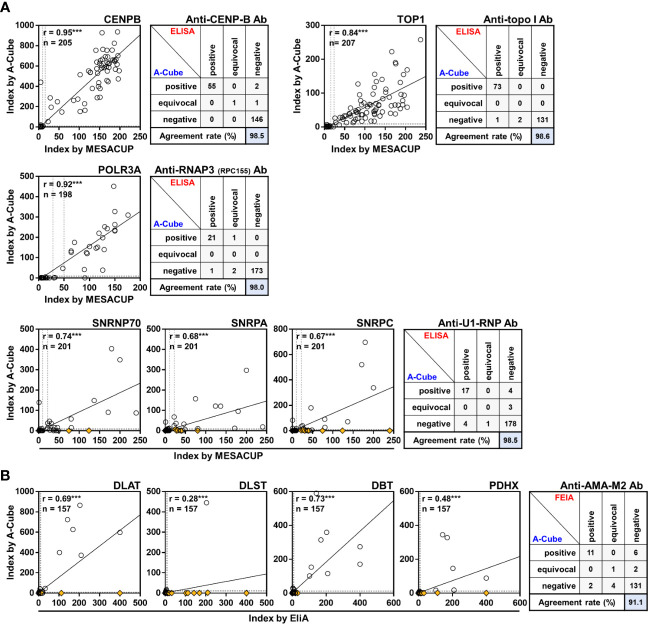
Correlation with conventional ELISA. Autoantibody levels were compared between A-Cube and commercially-available ELISA **(A)** and FEIA **(B)** kits. Dotted lines indicate the cut-offs for positive/equivocal/negative results. Yellow diamonds represent samples with reactivity with other autoantigens. Ab, antibody; ELISA, enzyme-linked immunosorbent assay; FEIA, fluoroenzyme immunoassay; RNAP3, RNA polymerase III; topo I, topoisomerase I.

We next compared the results of A-Cube with those obtained by ELISA and FEIA kits that were well established and approved by the regulatory authority in Japan. There was a significant positive correlation between the autoantibody index by A-Cube and the ELISA kits for anti-CENP-B antibody (r = 0.95, p< 0.001), anti-topoisomerase I-antibody (r = 0.84, p< 0.001), and anti-RNA polymerase III (RPC155) antibody (r = 0.92, p< 0.001; **Figure 3A**). The agreement rates between A-Cube and the ELISA kits were also high with 98.5%, 98.6%, and 98.0% for anti-CENP-B antibody, anti-topoisomerase I antibody, and anti-RNA polymerase III (RPC155) antibody, respectively. In addition, A-Cube detected each target antigen of anti-U1-RNP antibodies (SNRP70, SNRPA, and SNRPC) while achieving a high agreement rate (98.5%) with the ELISA kit that uses the mixture of these antigens and therefore cannot distinguish between them. Similarly, A-Cube recognized each of the four target antigens of anti-AMA-M2 antibody (DLAT, DLST, DBT, and PDHX) while showing a high agreement rate (91.1%) with the FEIA kit (**Figure 3B**). The Cohen’s kappa coefficients were 0.972 for anti-CENP-B antibody, 0.984 for anti-topoisomerase I antibody, 0.956 for anti-RNA polymerase III (RPC155) antibody, 0.765 for anti-U1-RNP antibody, and 0.665 for anti-AMA-M2 antibody, indicating a high concordance between A-Cube and ELISA/FEIA kits for these autoantibodies. The results of A-Cube and ELISA kits also showed high consistency for myositis-associated autoantibodies, with the Cohen’s kappa coefficient being 0.878 for anti-ARS antibody, 1.000 for anti-Mi-2 antibody, and 0.799 for anti-TIF1-γ antibody. Collectively, A-Cube not only provided consistent results with established ELISA and FEIA kits, but also allowed the evaluation of different target antigens of some autoantibodies.

### Validation by IP

**Figure 4:**
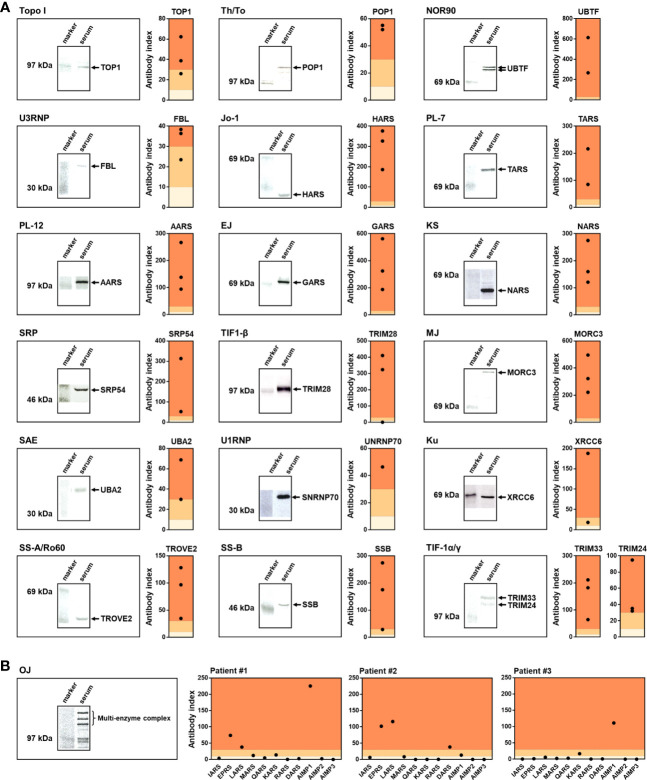
Validation by immunoprecipitation. **(A, B)** Serum samples validated by IP were analyzed by A-Cube. The left shows the representative autoradiographs of 7.5% SDS-polyacrylamide gel electrophoresis of immunoprecipitates. The right shows the index of antibody evaluated by A-Cube. The background of the graph was colored according to the threshold; dark orange = positive, pale orange = equivocal, and ivory = negative.

Subsequently, we compared the results of A-Cube with those of IP, the gold standard for autoantibody detection (**Figures 4A, B**). A total of 49 serum samples that were validated for 20 autoantibodies by IP were used for the analysis. A-Cube detected the expected autoantibodies in 98% (48/49) of these samples, showing its high sensitivity. Of note, A-Cube successfully detected anti-OJ antibody in 3 of 3 validated samples by evaluating all components of the multi-enzyme synthetase complex (IARS, EARS, LARS, MRAS, QARS, KARS, RARS, DARS, AIMP1, AIMP2, and AIMP3; **Figure 4B**). Indeed, all three samples showed reactivity with components other than IARS, the presumed major target antigen of anti-OJ antibody (19). This result is consistent with the weak antigenic activity of IARS and the difficulty of detecting anti-OJ antibody by conventional ELISAs and immunoblotting assays that carry only IARS as the target antigen (20). Overall, A-Cube showed high agreement rates with IP for various autoantibodies, in part by covering a wide range of target antigens.

### Autoantibody landscape of SSc and PM/DM

**Figure 5:**
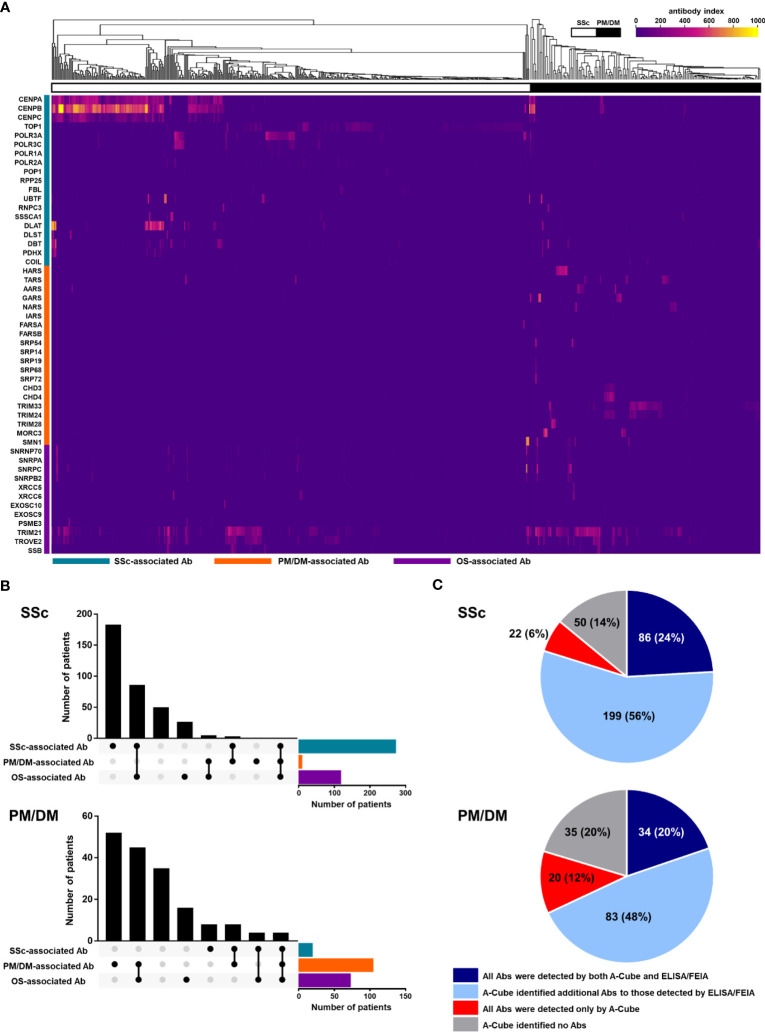
Autoantibody landscape in SSc and PM/DM. **(A)** Heatmap of autoantibodies in 357 SSc and 172 PM/DM patients. **(B)** The UpSet plot of autoantibodies in patients with SSc and PM/DM. **(C)** Pie chart representing the number of autoantibodies detected by A-Cube and conventional ELISA/FIEA kits. Ab, antibody; ELISA, enzyme-linked immunosorbent assay; FEIA, fluoroenzyme immunoassay; OS, overlap syndrome; PM/DM, polymyositis/dermatomyositis; SSc, systemic sclerosis.

Following the validation of A-Cube, we explored the autoantibody landscape in patients with SSc (n = 357) and PM/DM (n = 172; **Figure 5A**). In SSc, 76.5% (273/357) of the patients were positive for SSc-associated autoantibodies, and 24.1% (86/357), 0.8% (3/357), and 0.3% (1/357) also presented with overlap syndrome-associated autoantibodies, PM/DM-associated autoantibodies, and both of these autoantibodies, respectively. In PM/DM, 61.0% (105/172) of the patients were positive for PM/DM-associated autoantibodies, and 26.2% (45/172), 2.3% (4/172), and 2.3% (4/172) also presented with overlap syndrome-associated autoantibodies, SSc-associated autoantibodies, and both of these autoantibodies, respectively (**Figure 5B**). We also compared the detection rates of autoantibodies by A-Cube and conventional ELISA/FEIA kits that were approved by the regulatory authority in Japan (**Figure 5C**). A-Cube identified at least one autoantibody in 86.0% (307/357) of SSc and 79.7% (137/172) of PM/DM patients. Among them, 6.2% (22/357) of SSc and 11.6% (20/172) PM/DM patients had a set of autoantibodies that cannot be detected by conventional ELISA/FEIA kits. Moreover, in 55.7% (199/357) of SSc and 48.3% (83/172) of PM/DM patients, A-Cube revealed additional autoantibodies to those detected by conventional ELISA/FEIA. Autoantibodies were detected only by the conventional ELISA/FEIA kits in 1.1% (4/357) of SSc and 1.2% (2/172) of PM/DM patients. Taken together, A-Cube significantly improved the detection rate of autoantibodies. Although the autoantibody profile was not validated by IP assays in all samples, these data suggest that A-Cube may be useful to detect a wide range of autoantibodies, including those not covered in conventional ELISA/FEIA kits.

### Overlap of multiple autoantibodies

**Figure 6:**
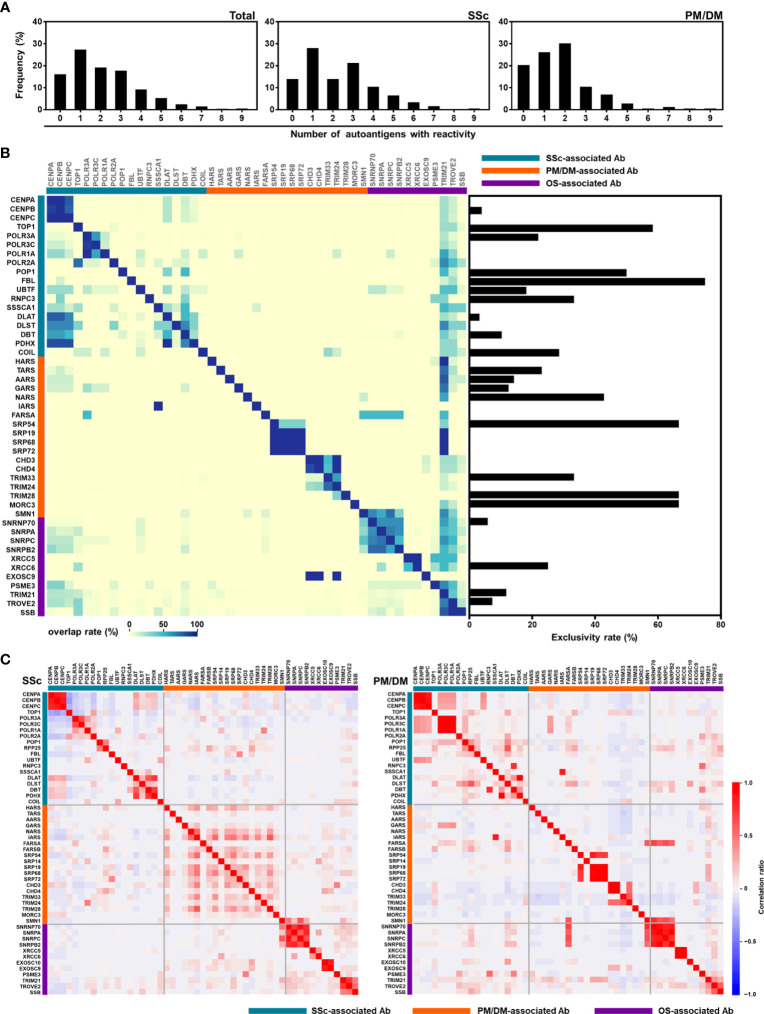
Overlapping of autoantibodies. **(A)** Number of autoantibodies in patients with SSc (n = 357) and PM/DM (n = 172). **(B)** Heatmap representing the coexistence of autoantibodies (left) and exclusivity rate of autoantibodies (right). **(C)** Correlational heat map between each autoantibody in patients with SSc and PM/DM. Ab, antibody; OS, overlap syndrome; PM/DM, polymyositis/dermatomyositis; SSc, systemic sclerosis.

To dissect the heterogeneity of autoantibody profiles, we next studied the overlap of different autoantibodies in patients with SSc (n = 357) and PM/DM (n = 172). A-Cube detected two or more autoantibodies in 58.0% (207/357) of SSc and 53.5% (92/172) of PM/DM patients (**Figure 6A**). As shown in **Figure 6B**, coexistence of autoantibodies was frequently seen within those targeting the same protein group, such as anti-CENP antibodies (CENP-A, -B, and -C) and anti-Mi-2 antibodies (CHD3 and CHD4). In contrast, anti-topoisomerase I antibody and anti-U3-RNP antibody, anti-TIF1-β antibody, and anti-MJ (NXP-2) antibody were less likely to coexist with other autoantibodies, with the exclusive rate of 58.3% (56/96), 75.0% (6/8), 66.7.% (2/3), and 66.7% (4/6), respectively. The correlation matrix of autoantibody index showed mutual exclusivity between anti-CENP antibodies, anti-topoisomerase I antibody, and anti-RNA polymerase III antibodies in patients with SSc. Similarly, anti-ARS antibodies and anti-TIF1 antibodies were mutually exclusive in patients with PM/DM (**Figure 6C**). These data are consistent with previous studies of autoantibody profiles in SSc and PM/DM (3, 4). It is also noteworthy that coexistence of autoantibodies and positive correlations between autoantibody indices were more pronounced in SSc than in PM/DM, which might suggest the high immunological heterogeneity of SSc (21–23).

### Clinical significance of autoantibody profile revealed by A-Cube

**Figure 7:**
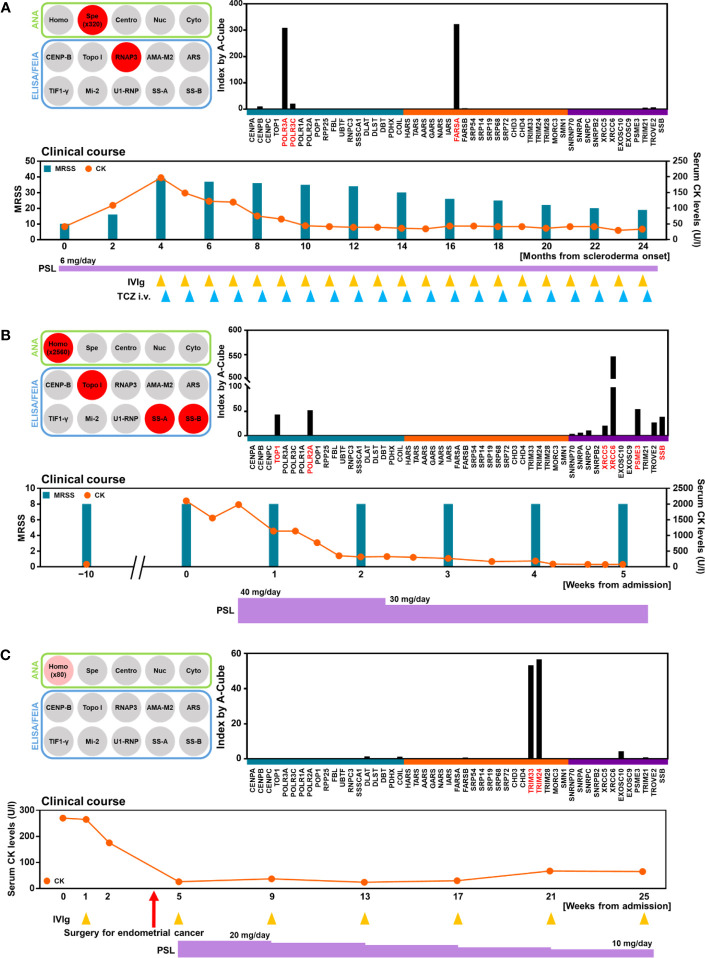
Autoantibody profile and clinical association. Autoantibody profiles and clinical courses were illustrated in three patients. **(A)** Detection of anti-Zo antibody by A-Cube. A 71-year-old woman with SSc and myositis showing the overlapping of anti-RNA polymerase III antibody and anti-Zo antibody. **(B)** Detection of multiple autoantibodies. A 55-year-old woman with SSc and myositis showing the overlapping of anti-topoisomerase I antibody, anti-U1-RNP_C antibody, anti-Ku antibody, and anti-Ki antibody. **(C)** Detection of anti-TIF1-γ and anti-TIF1-α antibodies in a 60-year-old woman with malignancy-associated DM. CK, creatinine kinase; DM, dermatomyositis; i.v., intravenous; IVIg, intravenous immunoglobulin; MRSS; modified Rodnan skin score; PSL, prednisolone; RNAP3, RNA polymerase III; TCZ, tocilizumab; topo I, topoisomerase I.

Lastly, we reviewed the clinical course of three patients whose autoantibody profiles were primarily revealed by A-Cube. The first case is a 71-year-old woman with rheumatoid arthritis and systemic lupus erythematosus controlled with oral prednisolone 6 mg/day (**Figure 7A**). She presented with skin sclerosis and proximal muscle weakness. Although SSc-myositis overlap was suspected, conventional ELISA only detected anti-RNA polymerase III (RPC155) antibody, and PM/DM-associated autoantibodies including anti-ARS antibodies were all negative. A-Cube revealed anti-Zo antibody, a rare member of anti-ARS antibodies that cannot be examined by conventional ELISA for anti-ARS antibodies (24), which only detects anti-Jo-1, anti-PL-7, anti-PL-12, anti-EJ, and anti-KS antibody (20). Further studies revealed elevated serum creatinine kinase (CK) levels and interstitial lung disease, both of which were compatible with anti-ARS syndrome (24). Combined immunosuppressive therapy including tocilizumab and intravenous immunoglobulin relieved the symptoms. The second case is a 55-year-old woman with a 20-year history of SSc (**Figure 7B**). She presented with fever, muscle weakness, and elevated serum CK levels. In addition to anti-topoisomerase I antibody and anti-SS-B antibody detected by conventional ELISA, A-Cube revealed anti-RNA polymerase II antibody, anti-U1RNP_C antibody, anti-Ku antibody, and anti-Ki antibody. Among them, anti-U1RNP_C antibody, anti-Ku antibody, and anti-Ki antibody supported the overlap of myositis with SSc (25–28). Following the systemic evaluation, myositis was successfully treated with oral prednisolone 40 mg/day. The third case is a 60-year-old woman who presented with proximal muscle weakness and pruritic skin eruption (**Figure 7C**). While PM/DM-associated autoantibodies including anti-TIF1-γ antibody were negative by conventional ELISA, A-Cube detected anti-TIF1-γ antibody and anti-TIF1-α antibody. In line with the previous studies reporting the association between the coexistence of these autoantibodies and malignancy (29), further evaluation revealed endometrial cancer. The myositis improved significantly following the laparotomy for endometrial cancer, supporting the close association between myositis and malignancy in the case. Collectively, these cases highlighted the utility of A-Cube in clinical settings.

## Discussion

In this study, we developed A-Cube, a multiplex wet protein array covering 65 target antigens of 43 autoantibodies that are associated with SSc and PM/DM (**Table 1**; **Figures 1**, **2**). A-Cube achieved a high agreement rate with IP and established ELISA kits, validating its reliability in autoantibody detection (**Figures 3**, **4**). Analysis of the serum samples by A-Cube meticulously illustrated the diverse landscape of autoantibodies in SSc and PM/DM (**Figures 5**, **6**). In clinical settings, A-Cube revealed multiple autoantibodies that cannot be detected by conventional ELISA, contributing to the diagnosis and proper management of SSc and PM/DM (**Figure 7**).

Thorough understanding of autoantibody profiles is an important challenge in the treatment of SSc and PM/DM, which are prototypical systemic autoimmune disorders with high heterogeneity (30, 31). In the past decades, an increasing number of autoantibodies have been identified in SSc and PM/DM, leading to the classification of the patients into more homogenous subgroups (3, 4). However, only a few major autoantibodies can be examined on a regular basis: the rest are often overlooked or underestimated. In our cohort, the wide coverage and high sensitivity of A-Cube (**Table 1**; **Figure 4**) markedly improved the detection of autoantibodies in patients with SSc and PM/DM (**Figure 5C**), enabling better management of these diseases (**Figure 7**). Moreover, accumulation of the data by A-Cube would further promote the characterization of these autoantibodies with unprecedented resolution.

Multiplex autoantibody detection is another important feature of A-Cube. Frequent coexistence of autoantibodies revealed by A-Cube (**Figures 5**, **6**) suggests that the overlap among autoantibodies should be further explored in patients with SSc and PM/DM. In particular, A-Cube successfully detected the coexistence of autoantibodies targeting the same protein group as well as those associated with overlap syndrome, both of which are shown to have clinical relevance in SSc and PM/DM (32–34). Moreover, recent studies have suggested that the number of autoantibodies itself can serve as a marker of autoreactivity in various autoimmune and inflammatory conditions (35–37), which is in line with our cases carrying multiple autoantibodies that required intensive immunosuppressive therapy (**Figures 7A, B**).

The major limitation of this study is the lack of detailed clinical information of each patient. Future studies of A-Cube with even more patients, broader clinical information, along with longitudinal data, will further advance our understanding of autoantibodies and their implications in SSc and PM/DM. Such studies should be ideally performed in samples collected from a large cohort, which would provide a robust database of comprehensive autoantibody profiles in these diseases. Another limitation of this study is that only a part of the results of A-Cube was validated by IP assays (**Figure 4**). Due to the shortage of serum samples, we could only perform IP assays for a limited number of autoantigens using two or three samples, depending on the number of samples with sufficient volume. Although the established ELISA/FEIA kits supported the reliability of A-Cube for several autoantibodies (**Figure 3**), all samples should be ideally validated by IP assays. In particular, some autoantibodies including anti-SAE, anti-Ku, anti-PM-Scl, and anti-cN1A antibodies were rarely detected in this study, which might be attributed to the tendency that patients with these autoantibodies usually have mild or no rashes and are less likely to visit a dermatologist, rather than a neurologist (4). In future studies, the accuracy of these rarely-detected autoantibodies should be also validated against IP assays. The comparison between the results of A-Cube and line blot assays would also provide insightful information about the utility of A-Cube in daily practice. In addition, A-Cube lacks the autoantigens for anti-MDA5 and anti-HMGCR antibodies due to patent issues, which limits the usefulness of the assay. While no patients fulfilled the criteria for clinically amyopathic DM antibodies (15) or were clinically suspected of having statin-induced myositis (16) in our cohort, the prevalence of myositis-specific autoantibodies in this study should be cautiously interpreted. Nevertheless, this study highlights the advantages, limitations, and potential of A-Cube in clinical and research settings, extending our previous work on HuPEX (38).

In summary, we developed A-Cube, a multiplex autoantibody assay with good reliability, high throughput, and wide coverage, by using a proteomic technique. Comprehensive analysis of serum samples by A-Cube uncovered a detailed autoantibody landscape with clinical significance in SSc and PM/DM. Although additional evaluation is needed to fully validate its performance, this study suggests that A-Cube has the potential to bring a paradigm shift in the way we diagnose, monitor, and treat autoimmune disorders.

## Data availability statement

The raw data supporting the conclusions of this article are not readily available because of participant privacy. The datasets are available from the corresponding authors upon reasonable request.

## Ethics statement

The studies involving humans were approved by the ethics committee of the University of Tokyo Graduate School of Medicine. The studies were conducted in accordance with the local legislation and institutional requirements. The participants provided their written informed consent to participate in this study.

## Author contributions

AK: Methodology, Investigation, Data curation, Formal Analysis, Writing – original draft. YN: Methodology, Investigation, Data curation. KM: Data curation. CO: Investigation. TO: Investigation. EK: Investigation. NG: Methodology, Resources. TF: Data curation. NF: Investigation. MH: Investigation. TY: Data curation. AY-O: Data curation. KY: Methodology, Writing – review & editing. TM: Data curation, Resources. SS: Conceptualization, Supervision, Writing – review & editing. AY: Conceptualization, Project administration, Supervision, Writing – review & editing.
